# Genome Assembly of a Putative Plant Growth-Stimulating Bacterial Sweet Pepper Fruit Isolate, Enterobacter hormaechei SRU4.4

**DOI:** 10.1128/mra.01237-22

**Published:** 2023-01-24

**Authors:** Tshifhiwa Paris Mamphogoro, Casper Nyaradzai Kamutando, Martin Makgose Maboko, Olubukola Oluranti Babalola, Olayinka Ayobami Aiyegoro

**Affiliations:** a Gastro-Intestinal Microbiology and Biotechnology Unit, Agricultural Research Council-Animal Production, Irene, Pretoria, South Africa; b Department of Plant Production Sciences and Technologies, University of Zimbabwe, Mount Pleasant, Harare, Zimbabwe; c Department of Crop Sciences, Tshwane University of Technology, Pretoria, South Africa; d Food Security and Safety Focus Area, Faculty of Natural and Agricultural Sciences, North-West University, Mmabatho, South Africa; e Research Unit for Environmental Sciences and Management, North-West University, Potchefstroom, South Africa; Loyola University Chicago

## Abstract

Here, we report the draft genome sequence of Enterobacter hormaechei SRU4.4. This bacterium (genome size = 4,440,516 bp; coding sequences = 4,100; G+C content = 56%) encodes for genes attributed to plant growth promotion (PGP).

## ANNOUNCEMENT

Enterobacter species are Gram-negative, facultative anaerobes known to colonize tomato fruit surfaces and other plant organs (1, 2). Several Enterobacter species harbor important plant growth promotion (PGP) traits, including antagonism of pathogenic microbial strains (3, 4). Therefore, studying the genome of this bacteria may help predict the genes responsible for the aforementioned beneficial traits.

Enterobacter hormaechei strain SRU4.4, reported here, was isolated from the surface of sweet pepper fruit obtained from an experimental field of the Agricultural Research Council-Vegetable and Ornamental Plants (25°59′ S, 28°35′ E) in Pretoria, South Africa (5). For isolation of the bacteria, bacterial biofilms were recovered from the surfaces of pepper fruits using sterile swabs soaked in a solution containing 0.15 M NaCl and 0.1% (vol/vol) Tween 20 (6). The swabs were vortexed in sterile Eppendorf tubes containing saline solution, serial dilutions were plated in triplicate on Trypticase soy agar (TSA), and plates were incubated at 30°C for 48 h under aerobic conditions. Distinct bacterial colonies were selected based on their morphological appearance. Pure culture of the bacterial isolate was obtained by repeated streaking onto sterile TSA and stored in 50% (vol/vol) glycerol at −80°C for further use.

After 48 h of incubation, genomic DNA was extracted from scraped-off plate cultures in TSA, employing a Quick-DNA miniprep kit specific for fungi or bacteria (Zymo Research, Irvine, CA, USA; catalog number D6005), as described in reference 7. DNA concentration was measured using a NanoDrop instrument (Thermo Fisher Scientific, Carlsbad, CA, USA), while DNA quality was evaluated by electrophoretic separation on a 2% (wt/vol) agarose gel. SRU4.4 was taxonomically identified by amplifying its 16S rRNA gene as described in reference 8. Whole-genome sequencing was performed on the Illumina NextSeq 550 (2 × 150) platform at Inqaba Biotechnical Industries, South Africa. A DNA library was constructed using a NEBNext Ultra II FS DNA library preparation kit (New England Biolabs, Massachusetts, USA). A total of 2,213,723 150-bp paired-end reads, representing ~150× genome coverage, were generated.

Raw sequences were quality filtered on the KBase (9) platform. Sequence quality was checked using FastQC v0.11.5 (10). Low-quality reads and sequence adaptors were trimmed using Trimmomatic v0.36 (11). High-quality reads were *de novo* assembled using SPAdes v3.15.3 (12) with default parameters. Sequence analysis yielded a total of 35 contigs with *N*_50_ and *L*_50_ values of 195,629 bp and 9, respectively, a genome size of 4,440,516 bp, and a G+C content of 56%. Gene annotation and prediction were performed with RASTtk (Rapid Annotations using Subsystems Technology toolkit) v1.073 as well as by following the NCBI Prokaryotic Genome Annotation Pipeline (PGAP) v6.3 (13, 14). Annotation procedures predicted 4,100 protein-coding genes, 62 RNA genes, 1 rRNA gene, 53 tRNA genes, 26 pseudogenes, and 8 noncoding RNA genes. *In silico* analysis using antiSMASH v6.0.0 (15) and RAST revealed the presence of genes involved in nitrogen metabolism, indole acetate acetic acid (IAA) production, siderophore production, phosphate solubilization, and biofilm production (Table 1), all of which play a significant role in PGP (16, 17).

**TABLE 1:** Genome annotation of plant growth-promoting strain SRU4.4^*a*^

Pathway	Gene	Locus tag	Product^*a*^
Nitrogen metabolism	*glnS*	LMH90_013350	Glutamine-tRNA ligase
	*gltX*	LMH90_013995	Glutamate-tRNA ligase
	*gltP*	LMH90_016460	Glutamate/aspartate:proton symporter GltP
	*glnA*	LMH90_017810	Glutamate-ammonia ligase
	*glnH*	LMH90_018095	Glutamine ABC transporter substrate-binding protein GlnH
	*glnP*	LMH90_018100	Glutamine ABC transporter permease GlnP
	*glnQ*	LMH90_018105	Glutamine ABC transporter ATP-binding protein GlnQ
Dissimilatory nitrate reduction	*nirB*	LMH90_000510	Nitrite reductase large subunit NirB
	*narH*	LMH90_000545	Nitrate reductase subunit beta
	*narJ*	LMH90_000550	Nitrate reductase molybdenum cofactor assembly chaperone
	*narI*	LMH90_000555	Respiratory nitrate reductase subunit gamma
	*nirD*	LMH90_003845	Nitrite reductase small subunit NirD
Ammonia assimilation	*gltX*	LMH90_013995	Glutamate-tRNA ligase
	*gltP*	LMH90_016460	Glutamate/aspartate:proton symporter GltP
Phosphate solubilization	*phoU*	LMH90_003490	Phosphate signaling complex protein PhoU
	*ppx*	LMH90_012150	Exopolyphosphatase
Phosphate degradation	*phnC*	LMH90_016330	Phosphonate ABC transporter ATP-binding protein
	*phnD*	LMH90_016335	Phosphonate ABC transporter substrate-binding protein
	*phnE*	LMH90_016340	Phosphonate ABC transporter, permease protein PhnE
	*phnF*	LMH90_016345	Phosphonate metabolism transcriptional regulator PhnF
Phosphate transport	*pstB*	LMH90_003495	Phosphate ABC transporter ATP-binding protein PstB
	*pstA*	LMH90_003500	Phosphate ABC transporter permease PstA
	*pstC*	LMH90_003505	Phosphate ABC transporter permease PstC
	*pstS*	LMH90_003510	Phosphate ABC transporter substrate-binding protein PstS
	*ugpE*	LMH90_004135	*sn*-Glycerol-3-phosphate ABC transporter permease UgpE
	*ugpA*	LMH90_004140	*sn*-Glycerol-3-phosphate ABC transporter permease UgpA
	*agp*	LMH90_005795	Bifunctional glucose-1-phosphatase/inositol phosphatase
	*phoH*	LMH90_005910	Phosphate starvation-inducible protein PhoH
Iron(II) transport	*feoA*	LMH90_003975	Ferrous iron transporter A
	*feoB*	LMH90_003980	Fe^2+^ transporter permease subunit FeoB
	*feoC*	LMH90_003985	Fe-S-dependent transcriptional repressor FeoC
	*fetA*	LMH90_015215	Iron ABC transporter ATP-binding protein FetA
	*fetB*	LMH90_015220	Iron export ABC transporter permease subunit FetB
Enterobactin production	*entD*	LMH90_012840	Enterobactin synthase subunit EntD
	*entE*	LMH90_012895	(2,3-Dihydroxy benzoyl)adenylate synthase EntE
	*entA*	LMH90_012905	2,3-Dihydro-2,3-dihydroxybenzoate dehydrogenase EntA
	*entH*	LMH90_012910	Proofreading thioesterase EntH
Siderophore production	*entD*	LMH90_012840	Enterobactin synthase subunit EntD
	*fes*	LMH90_012850	Enterochelin esterase
l-Tryptophan; IAA production	*trpA*	LMH90_000720	Tryptophan synthase subunit alpha
	*trpB*	LMH90_000725	Tryptophan synthase subunit beta
	*trpCF*	LMH90_000730	Bifunctional indole-3-glycerol-phosphate synthase TrpC/phosphoribosylanthranilate isomerase TrpF
	*trpD*	LMH90_000735	Anthranilate phosphoribosyltransferase TrpD
	*aldA*	LMH90_002155	Aldehyde dehydrogenase
	*trpS*	LMH90_010130	Tryptophan-tRNA ligase
Cytokinin	*miaA*	LMH90_010760	Adenosine(37)-*N*6-dimethylallyltransferase MiaA
	*miaB*	LMH90_013275	*N*6-Isopentenyl adenosine(37)-*C*2-methylthiotransferase MiaB
Ammonia production	*nadE*	LMH90_014565	NAD^+^ synthetase
Biofilm production	*yhjD*	LMH90_004490	Inner membrane protein YhjD
	*hfq*	LMH90_010755	RNA chaperone Hfq
	*bcsG*	LMH90_020670	Cellulose biosynthesis (CP) protein BcsG
	*bcsF*	LMH90_020675	Cellulose biosynthesis protein BcsF
	*bcsR*	LMH90_020685	Cellulose biosynthesis protein BcsR
	*bcsQ*	LMH90_020690	Cellulose biosynthesis protein BcsQ
	*bcsA*	LMH90_020695	UDP-forming cellulose synthase catalytic subunit
	*bcsB*	LMH90_020700	CP cyclic di-GMP-binding regulatory protein BcsB
	*bcsZ*	LMH90_020705	Cellulose synthase complex periplasmic endoglucanase BcsZ
	*bcsC*	LMH90_020710	Cellulose synthase complex outer membrane protein BcsC

a CP, cellulose biosynthesis.

### Data availability.

This whole-genome shotgun project and associated data have been deposited at DDBJ/ENA/GenBank under the accession number JAODYG000000000, with BioProject accession number PRJNA771517, BioSample accession number SAMN22357974, and SRA accession number SRR16379970. The version described in this paper is version JAODYG020000000.
